# Molecular and clinical insights into HIV-associated and HIV-negative aggressive B-cell lymphomas: prognostic quantitative biomarker analysis and therapeutic implications

**DOI:** 10.3389/fonc.2025.1603801

**Published:** 2025-05-27

**Authors:** Liming Liu, Tianwa Wang, Jiping Luo, Jianrong Huang, Kaipeng Huang, Kun Chen, Chao Wang, Zhiqiang Cheng

**Affiliations:** ^1^ Department of Pathology, Shenzhen Third People’s Hospital (The Second Affiliated Hospital of Southern University of Science and Technology), Shenzhen, Guangdong, China; ^2^ Department of Pathology (Longhua Branch), Shenzhen People’s Hospital (The Second Clinical Medical College, Jinan University), Shenzhen, Guangdong, China; ^3^ Department of Nephrology, Shenzhen Third People’s Hospital (The Second Affiliated Hospital of Southern University of Science and Technology), Shenzhen, Guangdong, China; ^4^ Jiangsu Cancer Hospital (Cancer Hospital Affiliated to Nanjing Medical University & Jiangsu Institute of Cancer Prevention and Control), Nanjing, Jiangsu, China; ^5^ Nanjing Medical University The Fourth School of Clinical Medicine, Nanjing, Jiangsu, China; ^6^ Luohu Mental Rehabilitation Department, Shenzhen Mental Health Center/Shenzhen Kangning Hospital, Shenzhen, Guangdong, China

**Keywords:** HIV-associated lymphomas, aggressive B-cell lymphoma, Epstein-Barr virus, quantitative immunophenotyping, prognostic modelling

## Abstract

**Background:**

HIV-associated lymphomas (HALs) exhibit aggressive features and poorer prognosis compared to HIV-negative lymphomas. However, their molecular and clinicopathological characteristics remain unclear in the antiretroviral therapy (ART) era.

**Methods:**

We retrospectively analyzed 208 lymphoma patients (57 HALs, 151 HIV-negative lymphomas) diagnosed between July 2019 and March 2024. Quantitative immunohistochemistry evaluated expression levels of Ki67, CD10, BCL6, MUM1, BCL2, and MYC. Independent prognostic factors were identified using multivariate Cox regression analysis, and a survival prediction model was validated by receiver operating characteristic (ROC) curve analysis.

**Results:**

HALs exhibited significantly higher proliferative activity (Ki67 AOD: 0.92 vs. 0.82, P < 0.001), more advanced disease stages (Ann Arbor stage III/IV: 77.2% vs. 60.0%, P = 0.022), and increased Epstein–Barr virus (EBV) positivity (51.1% vs. 17.9%, P < 0.001). Immunophenotyping revealed a GCB-like phenotype in HALs, characterized by elevated CD10 and BCL6 expression and decreased MUM1 and BCL2 expression. Patients with HALs had significantly shorter survival (median: 32.1 vs. 46.1 months, P < 0.001). Multivariate analysis identified Ki67 AOD (hazard ratio [HR] = 3.04, 95% confidence interval [CI]: 3.85–10.85), International Prognostic Index (IPI) (HR = 9.35, 95% CI: 4.20–20.82), and ART duration (protective, HR = 0.29/year, 95% CI: 0.19–0.45) as independent prognostic factors. The survival model demonstrated strong predictive accuracy (1-year area under the curve [AUC] = 0.831).

**Conclusions:**

HALs exhibit distinct molecular profiles—including elevated EBV infection, a GCB-like phenotype, increased Ki67 AOD, and decreased BCL2 expression—that contribute to significantly poorer survival compared to HIV-negative lymphomas. Integrating Ki67 AOD and IPI scores into prognostic models may enhance individualized prognosis and optimize treatment strategies for HAL patients.

## Introduction

HIV-associated lymphomas (HALs) represent a highly aggressive subset of hematologic malignancies, exhibiting rapid tumor progression and significantly reduced survival compared to HIV-negative lymphomas (1). Despite advances in antiretroviral therapy (ART), which have improved overall survival in people living with HIV (PLWH), HALs remain a major clinical challenge due to their distinct molecular features, high tumor burden, and resistance to standard treatments. The reported median overall survival (OS) for HAL patients remains between 6 and 24 months, significantly shorter than that of HIV-negative lymphoma patients receiving equivalent therapy (2). Furthermore, HALs are diagnosed at advanced stages (Ann Arbor stage III–IV in >80% of cases), with extranodal involvement frequently observed in the central nervous system (CNS) (20%–40%), gastrointestinal tract (30%–50%), and bone marrow (30%–60%), all of which contribute to poorer prognoses (3, 4). A defining characteristic of HALs is their strong association with Epstein-Barr virus (EBV) infection, which is detected in approximately 50%–80% of HAL cases, compared to <20% in HIV-negative lymphomas (5). EBV serves as a critical oncogenic driver by upregulating MYC signaling, inhibiting apoptosis via BCL2 and NF-κB activation, and promoting immune evasion through PD-L1 overexpression (6). Notably, EBV-positive HAL cases exhibit higher proliferative indices, as evidenced by Ki67 expression exceeding 80% in most cases, compared to a median Ki67 index of 40%–60% in HIV-negative lymphomas. The influence of EBV on lymphoma progression suggests that viral status may serve as a prognostic biomarker and potential therapeutic target. Beyond viral factors, HALs and HIV-negative lymphomas also demonstrate significant differences in their immunophenotypic and molecular characteristics (7). Immunohistochemical profiling has shown that HALs more frequently exhibit a germinal center B-cell-like (GCB) phenotype, with reported rates of 50%–80%, whereas HIV-negative lymphomas predominantly display a non-GCB phenotype (50%–80%) (8). HALs also exhibit significantly higher expression of proliferation markers such as MYC (≥40% in 55% of cases) and BCL2 (≥50% in 65% of cases), meeting the criteria for double-expressor lymphoma (DEL) in a large proportion of cases (9). These molecular features contribute to the inferior response rates observed in HALs, particularly with standard immunochemotherapy regimens such as R-CHOP, where 3-year progression-free survival (PFS) remains below 40%, compared to 55%–70% in HIV-negative lymphomas (10, 11). Despite these distinct biological and clinical characteristics, current risk stratification models and treatment guidelines for HALs remain largely extrapolated from studies on HIV-negative lymphomas. Prognostic indices such as the International Prognostic Index (IPI) and age-adjusted IPI, while useful, do not account for HIV-specific factors such as CD4+ T-cell counts, ART status, or immune reconstitution dynamics, which may significantly influence treatment response and survival outcomes (12). A critical gap in HAL research is the lack of comprehensive comparative analyses integrating multiparametric immunohistochemical (IHC) profiling, digital pathology-based quantification of proliferative indices, and EBV-encoded RNA (EBER) *in situ* hybridization with machine learning-assisted risk stratification. To address this gap, we conducted a retrospective cohort study to systematically compare the clinicopathological and molecular characteristics of HALs and HIV-negative lymphomas. Our findings reveal that HALs exhibit unique molecular vulnerabilities, including enhanced dependence on EBV-mediated NF-κB activation and PD-1/PD-L1 immune checkpoint signaling, which are underrepresented in current therapeutic paradigms. By leveraging high-throughput proteomic profiling (Nanostring GeoMx^®^) and circulating tumor DNA (ctDNA) monitoring, we identify actionable targets for HAL-specific precision therapy, such as EBV-directed CAR T-cells or PD-1 blockade in combination with MYC inhibition. Our analysis focused on key parameters, including EBV infection status, quantitative immunophenotypic markers, proliferative indices, and survival outcomes, to identify prognostic biomarkers that could refine risk stratification and inform targeted therapeutic strategies for HAL patients. By integrating molecular and clinical data, this study aims to improve the understanding of HAL pathogenesis and contribute to the development of tailored treatment approaches to enhance patient outcomes.

## Materials and methods

Ethical approval for the study was obtained from the Ethics Committee of Shenzhen Third People’s Hospital (Approval No.: SZTPH-EC-2025-003). Since only de-identified clinical and pathological data were utilized, the requirement for informed consent was waived by the Ethics Committee Ethics Committee of Shenzhen Third People’s Hospital.

### Study design and patient enrollment

This retrospective cohort study included 208 patients diagnosed with lymphoma between July 2019 and March 2024 at Shenzhen Third People’s Hospital. Among the enrolled patients, 57 were diagnosed with HALs and 151 with HIV-negative lymphomas. A subgroup analysis was conducted for aggressive B-cell lymphomas—including diffuse large B-cell lymphoma (DLBCL), Burkitt lymphoma (BL), and high-grade B-cell lymphoma (HGBL)—comprising 43 HALs and 57 HIV-negative cases (13).

### Inclusion and exclusion criteria

**Figure 1:**
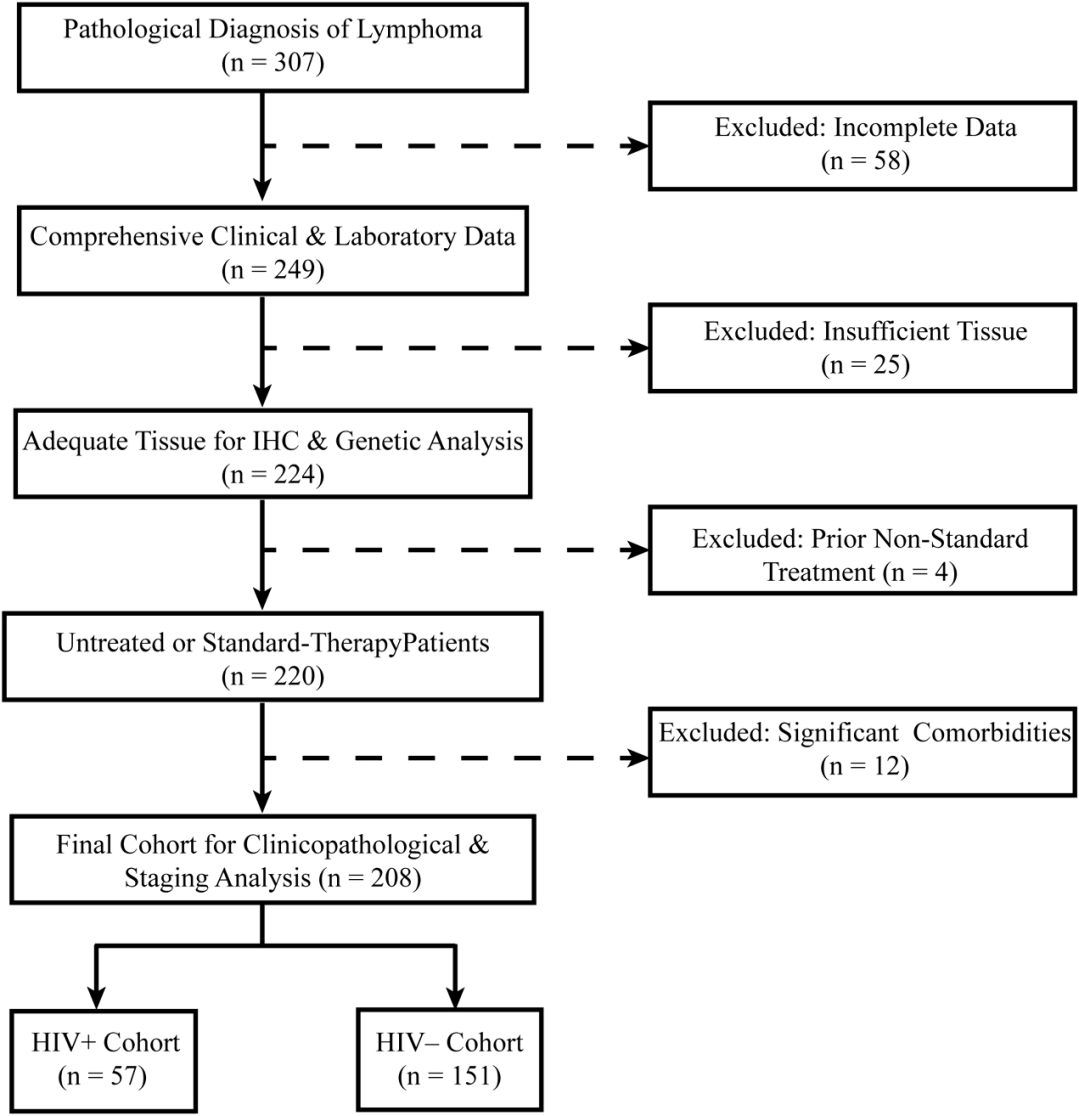
Flowchart illustrating the inclusion and exclusion criteria for patient selection.

Exclusion criteria included: Incomplete clinical or pathological data, Insufficient tissue samples for immunohistochemical (IHC) analysis, Prior non-standard treatments before enrollment, Presence of significant comorbidities potentially confounding lymphoma progression, including: Active immunosuppressive conditions, Untreated coexisting malignancies diagnosed within the past two years, History of solid organ transplantation, A flow diagram outlining patient selection is presented in **Figure 1**.

### Clinical and laboratory data collection

HIV-1 infection was diagnosed according to WHO-recommended guidelines. All cases were confirmed by both serological and molecular testing, following a double verification process by Shenzhen Third People’s Hospital and the Shenzhen Center for Disease Control and Prevention (CDC) before classification as HIV-positive. For confirmed HIV-positive patients, CD4+ T-cell counts (normal range: 500–1,500 cells/μL) and HIV RNA levels (with viral suppression defined as <500 IU/mL) were measured at the time of lymphoma diagnosis (or within one month prior if not available), and ART status (receiving ART vs. ART-naïve) along with duration were recorded. In addition, clinical data such as age, sex, Eastern Cooperative Oncology Group (ECOG) performance status, Ann Arbor stage, IPI score, tumor localization (nodal versus extranodal), and treatment history were documented. The evaluation of co-infections was conducted by determining hepatitis B virus (HBV) status using ELISA and hepatitis C virus (HCV) status using ELISA followed by PCR confirmation. Tumor burden was assessed by measuring lactate dehydrogenase (LDH) levels, with values exceeding 250 U/L defined as elevated.

### Pathological and immunohistochemical analysis

Histopathological diagnoses were performed by two independent pathologists following the WHO-HAEM5 classification. Tissue samples were processed as formalin-fixed, paraffin-embedded (FFPE) blocks, sectioned at 4 μm thickness, and stained with hematoxylin and eosin (H&E) for morphological assessment. Immunohistochemical (IHC) analysis was carried out to evaluate the expression of CD10, BCL6, MUM1, BCL2, MYC, and Ki67 using standardized staining protocols (antibody details are provided in **Supplementary Table 1**). Slide images of stained sections were digitized using the SQS-600P scanner (Shengqiang Technology), and quantitative image analysis was performed in Fiji/ImageJ. The Color Deconvolution algorithm was applied to isolate DAB staining, enabling precise measurement of marker expression. Expression levels were semi-quantified based on the average optical density (AOD), which reflects the mean pixel intensity in DAB-positive tumor regions and provides a semi-quantitative measure of staining intensity. Additionally, the extent of marker expression was assessed by calculating the positive area percentage, representing the proportion of the tumor area exhibiting DAB-positive staining. Lymphoma subtypes were classified using the Hans algorithm, which stratifies diffuse large B-cell lymphoma (DLBCL) into germinal center B-cell-like (GCB) and non-GCB subtypes. Double-expressor lymphoma (DEL) was defined as MYC expression of at least 40% in conjunction with BCL2 expression of at least 50%. Epstein–Barr virus (EBV) status was determined by EBER *in situ* hybridization (ISH-7001, Zhongshan Golden Bridge Biotechnology) performed on FFPE tumor tissues, with cases classified as EBV-positive if any tumor cells exhibited nuclear EBER staining.

### Statistical analysis

Comparisons between HALs and HIV-negative lymphoma patients were conducted using Chi-square tests for categorical variables and Wilcoxon rank-sum tests for non-normally distributed continuous variables, with normality assessed via the Shapiro-Wilk test. Homogeneity of variance was evaluated using Levene’s test, and non-parametric methods were applied when the assumptions of normality or homogeneity were violated. Survival analysis was performed using the Kaplan–Meier method, with differences assessed by the log-rank test. Prognostic factors were identified through Cox proportional hazards models with stepwise selection. Model performance was further evaluated using receiver operating characteristic (ROC) curve analysis, with area under the curve (AUC) values calculated at 1, 2, and 3 years; additionally, ten-fold cross-validation was employed to assess model stability and generalizability. In the HALs subgroup, univariate Cox regression analysis was performed for HIV-related variables, including CD4 count at diagnosis, ART status, and ART duration. All statistical analyses were conducted using SPSS (version 29.0) and R software (version 4.2.2).

## Results

### Baseline demographic and clinicopathologic features

A total of 208 lymphoma patients were included in the study, with 57 cases classified as HALs and 151 as HIV-negative lymphomas. The patient selection process is illustrated in **Figure 1**. Compared with HIV-negative lymphomas, HALs patients were significantly younger (median age, 41 [IQR, 33–50.5] vs. 53 [IQR, 36–65] years; p = 0.003) and predominantly male (93.0% vs. 55.6%; p < 0.001). In addition, HALs more frequently presented with advanced disease, as evidenced by a higher proportion of Ann Arbor stage III/IV cases (77.2% vs. 60.0%; p = 0.022), elevated serum lactate dehydrogenase levels (median, 315 vs. 283 U/L; p = 0.003), and IPI (score 4–5: 50.9% vs. 31.8%; p = 0.006). Moreover, Epstein–Barr virus co-infection was significantly more common in HALs compared with HIV-negative lymphomas (51.1% vs. 17.9%; p < 0.001). Among HALs, 93.0% received antiretroviral therapy (ART); approximately half were on ART at the time of lymphoma diagnosis, while the remainder-initiated treatment afterward. The median ART duration was 20 months (IQR, 9–48), with a median CD4+ T-cell count of 127 cells/μL (IQR, 63.5–264), and viral suppression (HIV RNA <500 IU/mL) was achieved in 43.9% of cases (**Table 1**).

**Table 1:** Comparison of clinical features and pathological characteristics in lymphoma cases between (HALs and HIV-negative lymphomas).

Variable	HIV^-^ (n = 151)	HIV^+^ (n = 57)	*P* Value
Age, median (IQR), years	53 (36 - 65)	41 (33 - 50.5)	0.003*
Sex: no. (%)			
Male	84 (55.63)	53 (92.98)	< 0.001†
Female	67 (44.37)	4 (.072)	
LDH, median (IQR), U/L	283 (170 - 481)	315 (214.5 - 1244)	0.003*
Stage: no. (%)			
I/II	56 (40.0)	13 (22.81)	0.022*
III/IV	84 (60.0)	44 (77.19)	
NA	11	0	
IPI score: no. (%)			
0-1	34 (25.76)	7 (12.28)	0.006*
2-3	56 (42.42)	21 (36.84)	
4-5	42 (31.82)	29 (50.88)	
NA	19	0	
Tumor localization: no. (%)			
Nodal	85 (56.29)	30 (52.63)	0.636†
Extra nodal	66 (43.71)	27 (47.37)	
EBER: no. (%)			
Positive	22 (17.89)	23 (51.11)	< 0.001†
Negative	101 (82.11)	22 (48.89)	
NA	28	12	
HBV infection status: no. (%)			
PHI	55 (40.74)	17 (29.82)	0.153†
NHHI	80 (59.26)	40 (70.18)	
NA	16	0	
Diagnose: no. (%)			
B-cell	121 (80.13)	54 (94.94)	0.01†
T/NK cell	30 (19.87)	3 (5.26)	
Treatment for lymphoma: no. (%)			
Yes	99 (91.67)	36 (72.0)	0.001†
No	9 (8.33)	14 (28)	
NA	43	7	
Follow-up: no. (%)			
AWD	105 (75.54)	29 (50.88)	< 0.001†
DOD	34 (24.46)	28 (49.12)	
LFU	12	0	
Mean follow-up in months (95%CI)	46.12 (41.89 - 50.36)	32.12 (24.79 - 39.46)	< 0.001
ART for HIV: no. (%)	N/A	53 (92.98)	
ART at Lymphoma Diagnose	N/A	26 (49.06)	
ART after Lymphoma Diagnose	N/A	27 (50.94)	
ART time, median (IQR), months	N/A	20 (9 - 48)	
HIV-RNA <500 IU/mL, n (%)	N/A	25 (43.86)	
CD4+ T-cell count, median (IQR), cells/μL	N/A	127 (63.5 - 264)	
Ki67			
Median AOD, median (IQR)	0.82 (0.65 - 0.93)	0.92 (0.73 - 1.205)	< 0.001*
Median Positive Area (%), median (IQR)	61.36 (30.18 - 81.22)	85.87 (63.40 - 92.07)	< 0.001*

*Wilcoxon rank-sum test; †Chi-square test.

LDH, Lactate Dehydrogenase; IPI, International Prognostic Index; NA, Not Applicable; AWD, Alive with Disease; DOD, Dead of Disease; LFU, Lost to Follow-Up; ART, Antiretroviral Therapy; PHI, Persistent Hepatitis Infection; NHHI, Non-Hepatitis Hepatitis Infection.

### Survival analysis

**Figure 2:**
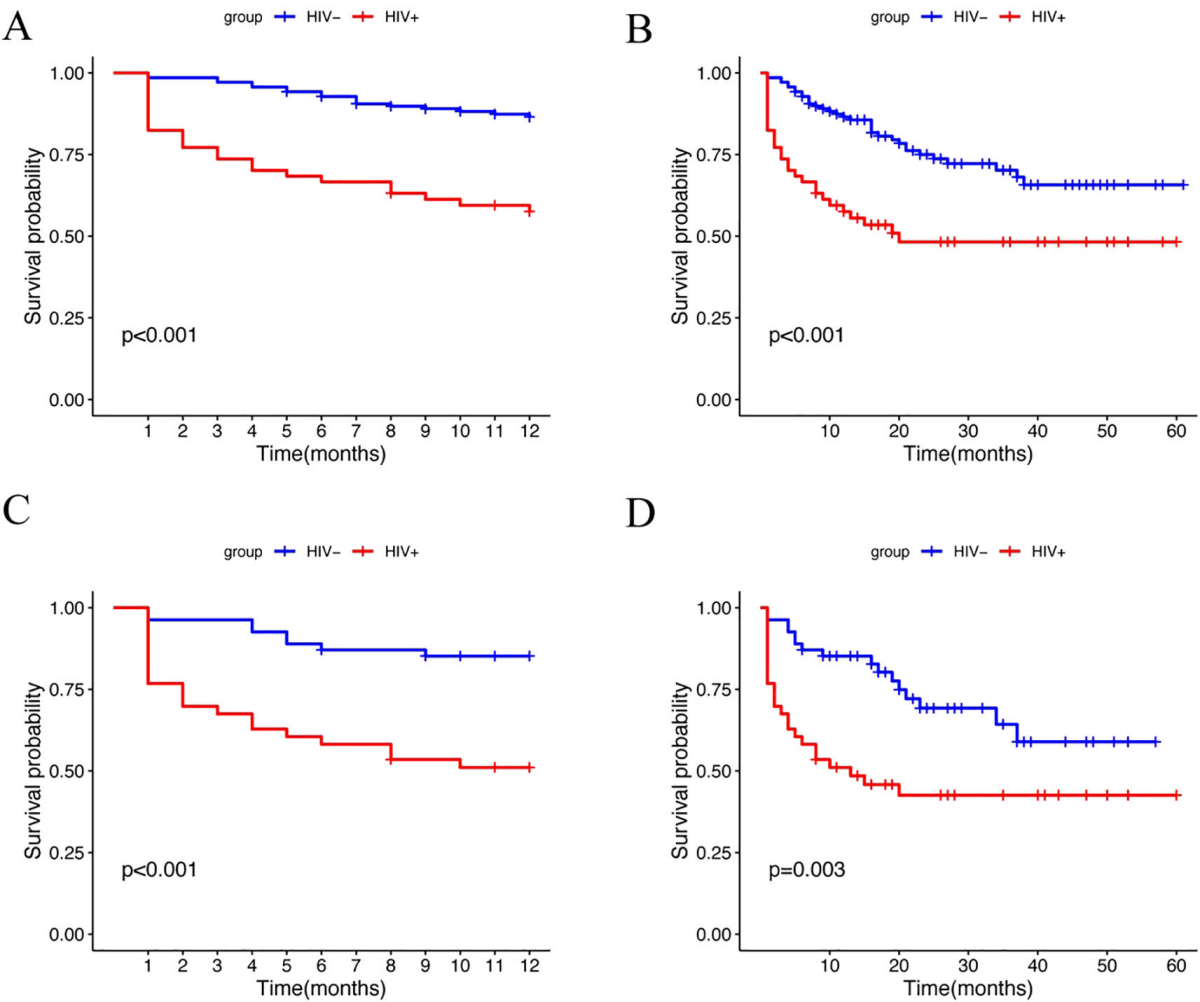
Kaplan-Meier survival analysis of lymphoma patients stratified by HIV status. **(A)** One-year survival curves for the HIV^+^ and HIV^–^ groups (log-rank test, p<0.001). **(B)** Overall survival curves for the HIV^+^ and HIV^–^ groups (log-rank test, p<0.001). **(C)** One-year survival curves for the HIV^+^ and HIV^–^ groups in the aggressive B-cell lymphoma subgroup (log-rank test, p<0.001). **(D)** Overall survival curves for the HIV^+^ and HIV^–^ groups in the aggressive B-cell lymphoma subgroup (log-rank test, p=0.003).

The survival analysis revealed significant findings. The one-year survival rate was significantly lower for HALs compared to HIV-negative lymphomas (**Figure 2A**; log-rank test, p < 0.001). HALs had a significantly shorter median overall survival (32.12 months [95% CI, 24.79–39.46]) compared to HIV-negative lymphomas (46.12 months [95% CI, 41.89–50.36]; p < 0.001; **Figure 2B**). Additionally, the one-year survival curves for the aggressive B-cell lymphoma subgroup showed a significant difference, with HALs having a lower survival rate (**Figure 2C**; log-rank test, p < 0.001). The mortality rate was substantially higher in HALs (49.1% vs. 24.5%; p < 0.001). A similar survival disadvantage was observed in the aggressive B-cell lymphoma subgroup, where HALs had a median survival of 28.4 months compared to 41.0 months in HIV-negative lymphomas (p = 0.003; **Figure 2D**).

### Histopathological and immunophenotypic analysis

**Figure 3:**
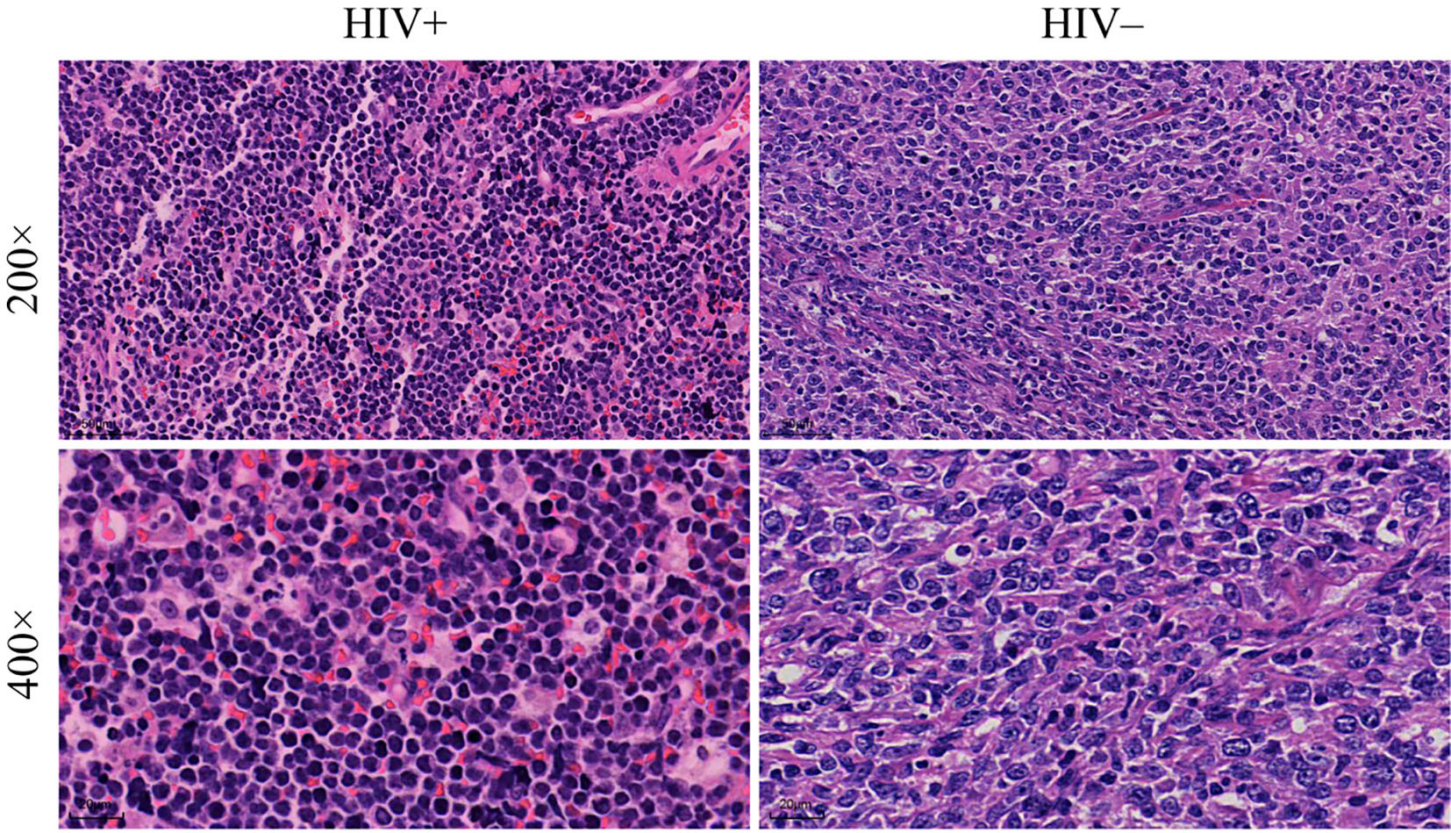
Histopathological analysis of lymphoma tissue samples. Comparison of tissue morphology between HIV^+^ and HIV^−^ patients. The images show lymphoma tissues at two magnifications: 200× (top row) and 400× (bottom row).

**Figure 4:**
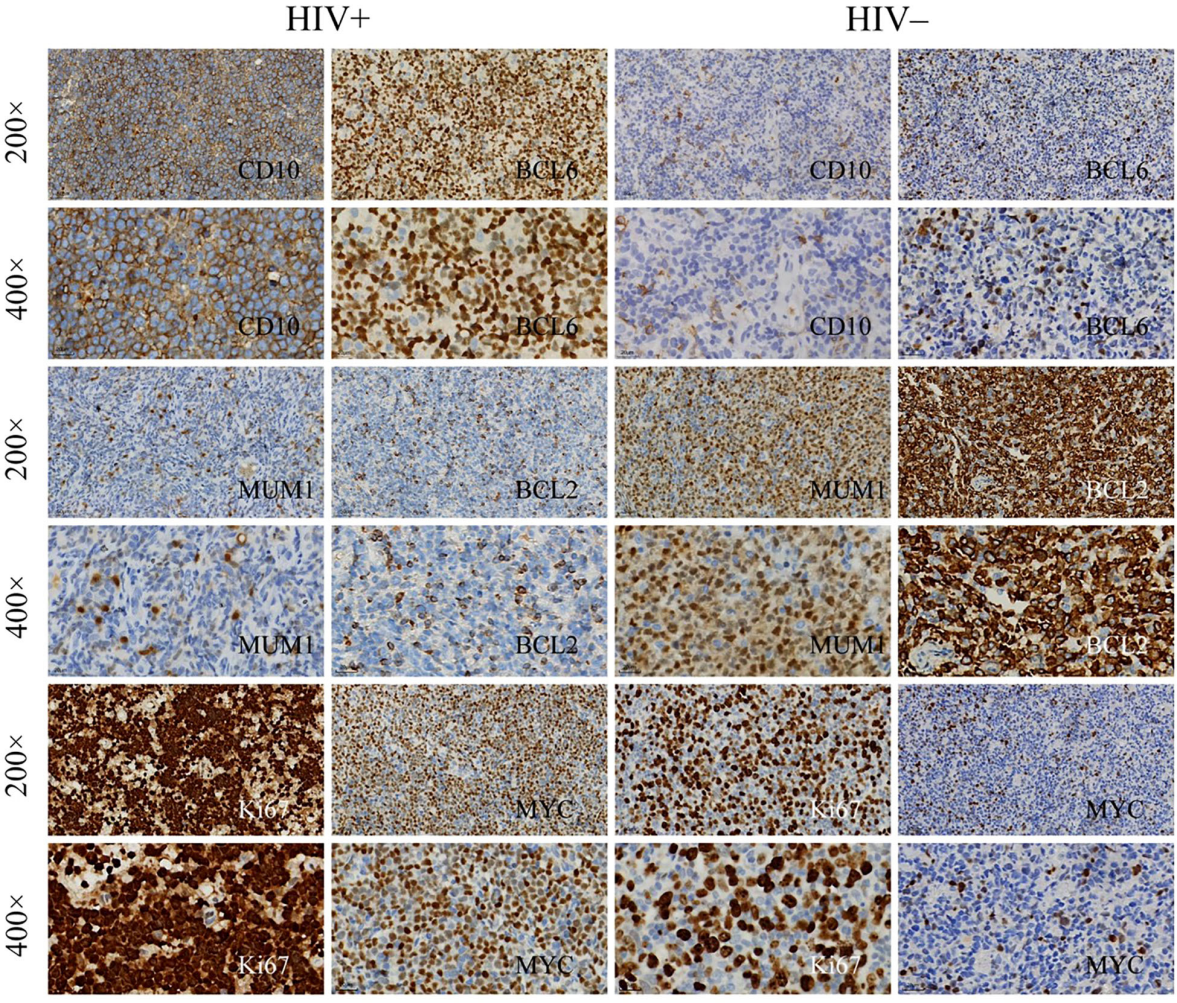
Immunohistochemical staining of lymphoma tissue samples. Immunohistochemical analysis of lymphoma tissues from HIV^+^ and HIV^−^ patients, showing expression of the following markets: Top rows: CD10 and BCL6 at 200× magnification and 400× magnification. Middle rows: MUM1 and BCL2 at 200×and 400×. Bottom rows: Ki67 and MYC at 200×and 400×.

In the histopathological evaluation, HALs showed more aggressive morphological features, including a diffuse growth pattern with near-complete effacement of normal lymph node architecture, more pronounced nuclear pleomorphism, and an increased frequency of mitotic figures. These observations suggest a higher degree of malignancy compared with HIV-negative lymphomas, as illustrated in representative H&E-stained sections (**Figure 3**). Differences in treatment exposure prior to tissue sampling could partially confound Ki67 comparisons; however, most biopsies were obtained before initiation of definitive chemotherapy, minimizing this effect. Immunohistochemical analysis revealed significant differences in biomarker expression between the groups. In the overall cohort, HALs exhibited higher Ki67 expression, with a median AOD of 0.92 (IQR, 0.73–1.205) versus 0.82 (IQR, 0.65–0.93) in HIV-negative lymphomas, and a greater median positive area percentage (85.87% vs. 61.36%; both p < 0.001; **Table 1**). In the aggressive B-cell lymphoma subgroup (**Table 2**), HALs showed significantly higher CD10 positivity (51.16% vs. 21.05%; p = 0.003) and elevated BCL6 expression, with a median AOD of 0.35 (IQR, 0.22–0.43) compared to 0.205 (IQR, 0.00–0.32) in HIV-negative lymphomas (p = 0.003), as well as a larger positive area percentage for BCL6 (49.35% vs. 13.95%; p = 0.002). Conversely, the expression of MUM1 and BCL2 was significantly lower in HALs (MUM1 positivity: 48.84% vs. 84.21%; p < 0.001 and BCL2 positivity: 51.16% vs. 78.95%; p = 0.007). MYC expression (assessed by AOD) was higher in HALs (0.29 vs. 0.265; p = 0.019). Moreover, Among DLBL cases, the Hans classification showed that HALs predominantly exhibited the GCB subtype (55.88%), whereas HIV-negative lymphomas were mainly classified as the non-GCB subtype (78.26%; p = 0.004). EBER positivity was also markedly increased among HALs (45.0% vs. 8.93%; p < 0.001), as shown in **Table 2** and **Figure 4**.

**Table 2:** Comparison of clinicopathological features of aggressive B-cell lymphomas in HIV- and HIV+ groups (HALs and HIV-negative lymphomas).

Variable	HIV^-^ (n = 57)	HIV^+^ (n = 43)	*P* Value
Age, mean (SD), years	53.88 (16.31)	44.09 (12.14)	0.001‡
Sex: no. (%)			
Male	36 (63.16)	39 (90.7)	0.002†
Female	21 (36.84)	4 (9.3)	
LDH, median (IQR), U/L	360 (210 - 491)	400 (272 - 1745)	0.097*
Stage: no. (%)			
I/II	16 (28.57)	5 (11.63)	0.15*
III/IV	40 (71.43)	38 (88.37)	
NA	1	0	
IPI score: no. (%)			
0-1	6 (10.71)	1 (2.33)	0.08*
2-3	26 (46.43)	16 (37.21)	
4-5	24 (42.86)	26 (60.47)	
NA	1	0	
Tumor localization: no. (%)			
Nodal	31 (54.39)	17 (39.53)	0.204†
Extra nodal	26 (45.61)	26 (60.47)	
HBV infection status: no. (%)			
PHI	26 (50.0)	10 (23.26)	0.014†
NHHI	26 (50.0)	33 (76.74)	
NA	5	0	
Diagnose: no. (%)			
DLBL	46 (80.7)	34 (79.07)	0.045†
BL	0	4 (9.3)	
HGBL	11 (19.3)	5 (11.28)	
Hans classification: no. (%)			
GCB	10 (21.74)	19 (55.88)	0.004†
Non-GCB	36 (78.26)	15 (44.12)	
DEL: no. (%)			
Yes	12 (21.05)	6 (13.95)	0.514†
No	45 (78.95)	37 (86.05)	
TEL: no. (%)			
Yes	7 (12.28)	3 (6.98)	0.590†
No	50 (87.72)	40 (93.02)	
Treatment for lymphoma: no. (%)			
Yes	38 (86.36)	28 (73.68)	0.123†
No	6 (13.64)	10 (26.32)	
NA	13	5	
HAART for HIV: no. (%)		40 (93.02)	
Follow-up: no. (%)			
AWD	38	19	0.003†
DOD	16	24	
LFU	3	0	
Median follow-up in months (95%CI)	40.99 (34.63 - 47.35)	28.41 (20.02 - 38.81)	0.003
Immunophenotype			
CD10			
Positive: no. (%)	12 (21.05)	22 (51.16)	0.003†
Negative: no. (%)	45 (78.95)	21 (48.84)	
Median AOD, median (IQR)	0.0 (0.0 - 0.0)	0.0 (0.0 -0.59)	< 0.001*
Median Positive Area (%), median (IQR)	0.0 (0.0 - 0.0)	0.0 (0.0 - 85.26)	< 0.001*
BCL6			
Positive: no. (%)	38 (66.67)	35 (81.4)	0.157†
Negative: no. (%)	19 (33.33)	8 (18.6)	
Median AOD, median (IQR)	0.205 (0.00 - 0.32)	0.35 (0.22 - 0.43)	0.003*
Median Positive Area (%), median (IQR)	13.95 (0.00 - 50.29)	49.35 (19.28 - 77.00)	0.002*
MUM1			
Positive: no. (%)	48 (84.21)	21 (48.84)	< 0.001†
Negative: no. (%)	9 (15.79)	22 (51.16)	
Median AOD, median (IQR)	0.38 (0.31 - 0.48)	0.0 (0.0 - 0.345)	< 0.001*
Median Positive Area (%), median (IQR)	57.61 (29.38 - 82.99)	0.0 (0.0 - 41.69)	< 0.001*
BCL2			
Positive: no. (%)	45 (78.95)	22 (51.16)	0.007†
Negative: no. (%)	12 (21.05)	21 (48.84)	
Median AOD, median (IQR)	0.425 (0.20 - 0.69)	0.16 (0.00 - 0.46)	0.001*
Median Positive Area (%), median (IQR)	62.71 (9.74 - 84.25)	9.45 (0.00 - 74.15)	0.003*
Ki67			
Median AOD, median (IQR)	0.895 (0.81 - 0.98)	1.00 (0.83 - 1.25)	0.005
Median Positive Area (%), median (IQR)	78.96 (61.5 - 90.29)	90.49 (81.03 - 92.48)	< 0.001
MYC			
Positive: no. (%)	41 (71.93)	37 (86.05)	0.149†
Negative: no. (%)	16 (28.07)	6 (13.95)	
Median AOD, median (IQR)	0.265 (0.03 - 0.31)	0.29 (0.23 - 0.42)	0.019*
Median Positive Area (%), median (IQR)	21.24 (0.68 - 38.98)	30.31 (8.89 - 53.78)	0.099*
EBER: no. (%)			
Positive	5 (8.93)	18 (45.0)	< 0.001†
Negative	51 (91.07)	22 (55.0)	
NA	1	3	

*Wilcoxon rank-sum test; †Chi-square test; ‡Student’s t-test.

LDH, Lactate Dehydrogenase; IPI, International Prognostic Index; NA, Not Applicable; AWD, Alive with Disease; DOD, Dead of Disease; LFU, Lost to Follow-Up; HAART, Highly Active Antiretroviral Therapy; PHI, Persistent Hepatitis Infection; NHHI, Non-Hepatitis Hepatitis Infection; DLBL, Diffuse Large B-Cell Lymphoma; BL, Burkitt Lymphoma; HGBL, High-Grade B-Cell Lymphoma; GCB, Germinal Center B-Cell; Non-GCB, Non-Germinal Center B-Cell; DEL, Double-Expressor Lymphoma; TEL, Triple-Expressor Lymphoma; AOD, Average Optical Density.

### Prognostic modeling

**Figure 5:**
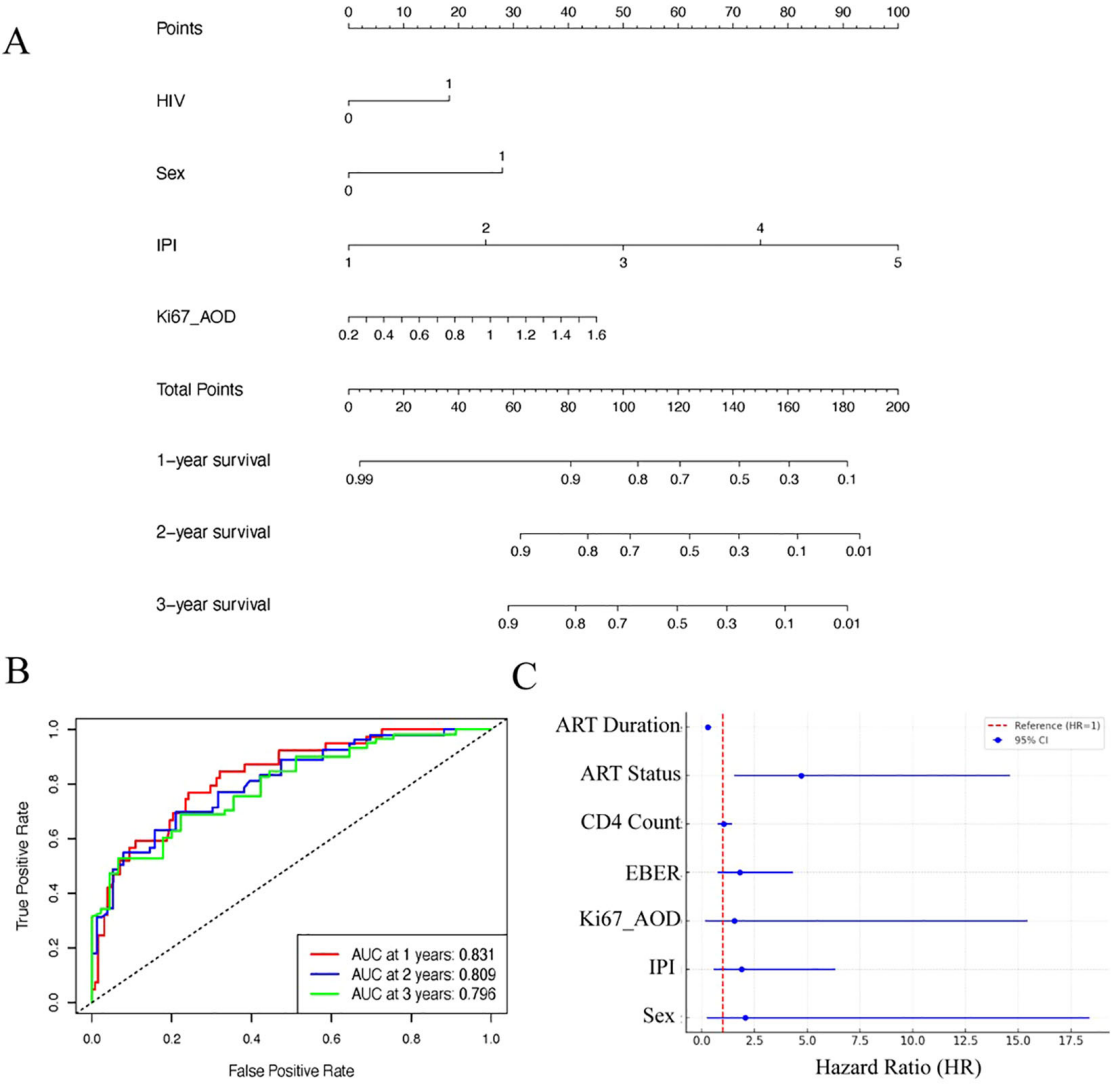
Prognostic modeling and analysis for lymphoma survival outcomes. **(A)** Nomogram based on clinical features and IHC markers for predicting 1-, 2-, and 3-year survival probabilities in lymphoma patients. **(B)** ROC curves showing the performance of the nomogram at different time points (1-, 2-, and 3-year survival). **(C)** Forest plot showing Hazard Ratios (HR) with their respective 95% confidence intervals (CI) for various prognostic factors associated with survival outcomes in HIV^+^ lymphomas.

Multivariate Cox regression analysis identified HIV infection (i.e., being in the HALs group; HR = 1.77; 95% CI, 1.05–3.01; p = 0.034), high IPI scores (HR = 9.35; 95% CI, 4.20–20.82; p < 0.001), and elevated Ki67 expression (assessed by AOD; HR = 3.04; p = 0.087) as independent predictors of poor overall survival (**Supplementary Table 2**). A prognostic nomogram (**Figure 5A**) incorporating these factors demonstrated robust predictive accuracy, with AUC values of 0.831, 0.809, and 0.796 for predicting 1-, 2-, and 3-year survival, respectively (**Figures 5B**). Ten-fold cross-validation was performed in a single iteration due to cohort size; future work will explore repeated cross-validation or bootstrap resampling to further assess model stability. (**Supplementary Figure 1**).

### Subgroup analysis of HALs

In a univariate Cox analysis within the HALs subgroup, longer ART duration was strongly associated with reduced mortality (HR = 0.29 per additional year; p < 0.001), highlighting the protective impact of sustained therapy. Although patients receiving ART at the time of lymphoma diagnosis exhibited a higher mortality risk (HR = 4.72; p = 0.007) compared with those who initiated ART after diagnosis, the duration of ART emerged as the most significant prognostic factor (**Figure 5C**).

## Discussion

Our study provides a comprehensive examination of the molecular and clinicopathologic differences between HALs and lymphomas in HIV-negative patients. This immunophenotypic pattern can be scientifically justified based on the pathogenesis of HIV-associated lymphomas. HIV infection leads to prolonged germinal center reactions and chronic immune activation, resulting in the expansion of B-cells with germinal center (GCB) phenotype. Therefore, high expression of CD10 and BCL6 and lower MUM1 expression reflects the predominance of GCB-type differentiation in HIV-related lymphomas, as supported by previous studies (14, 15). HAL patients in our cohort were younger and predominantly male, which reflects the underlying epidemiology of HIV infection, particularly in high-prevalence settings where younger men account for a disproportionate share of new HIV diagnoses. These demographic disparities likely influence both disease presentation and outcomes.We observed that HALs exhibit distinct biological characteristics that not only reflect differences in tumor pathogenesis but also have important prognostic and therapeutic implications. In our cohort, the rate of Epstein–Barr virus (EBV) infection was significantly higher in HALs compared with HIV-negative lymphomas, a finding that is consistent with prior reports from Ethopia (16). EBV plays a central role in driving oncogenesis in the context of immunosuppression, and its high prevalence in HALs is likely a consequence of HIV-induced CD4^+^ T-cell depletion (17). This immunosuppression weakens the host’s ability to control latent EBV infection, thereby facilitating the virus’s reactivation from latency to lytic replication. Specifically, LMP1 mimics constitutively active CD40 signaling, triggering NF-κB and PI3K/AKT cascades that promote B-cell proliferation, inhibit apoptosis, and enhance genomic instability. EBV-encoded proteins, such as latent membrane protein 1 (LMP1), can activate several oncogenic signaling pathways, including NF-κB and PI3K/AKT, which drive B-cell proliferation, promote genomic instability, and contribute to tumor progression (18). Our findings further indicate that the high EBV infection rate in HALs is associated with poorer survival, underscoring the adverse impact of EBV-driven oncogenesis in the immunocompromised setting. In addition to differences in viral status, the immunophenotypic profiles of HALs differ markedly from those of HIV-negative lymphomas. Specifically, HALs in our study demonstrated a predominance of the germinal center B-cell (GCB) phenotype, as evidenced by higher expression of CD10 and BCL6 and lower expression of MUM1. Approximately 55.88% of HIV-associated diffuse large B-cell lymphoma (DLBCL) cases were classified as GCB, whereas the majority of lymphomas in HIV-negative patients belonged to the non-GCB subtype. The molecular basis for this GCB predominance in HALs may relate to the impact of chronic HIV infection on B-cell development and differentiation. The restricted transition- germinal center B cells to post-germinal center memory B cells or plasma cells to post-germinal center differentiation observed in HALs likely reflects the altered cytokine milieu and chronic antigenic stimulation in the HIV-infected host. Clinically, the predominance of the GCB subtype in HALs carries potential therapeutic implications. In the general population, GCB-type DLBCLs tend to respond more favorably to standard regimens such as R-CHOP (19). However, the benefits of this immunophenotypic advantage in HALs may be mitigated by the added complexities of HIV-related immunosuppression and increased susceptibility to infections. This dichotomy highlights the need for tailored treatment strategies that account for both the tumor biology and the host’s immune status (20).

Notably, our finding of lower BCL2 expression in HALs contrasts with some *in vitro* studies, such as Castro-Gonzalez et al. (2021) (21), which demonstrated that HIV-1 Nef can enhance BCL2 function by promoting its association with BECN1 to inhibit autophagy. While those studies—conducted using established cell lines (e.g., HEK293T cells)—suggest a Nef-mediated upregulation of BCL2 to support viral replication, our clinical data indicate that HALs exhibit reduced BCL2 expression despite high proliferative activity, as evidenced by elevated Ki67 levels. A plausible explanation for this paradox is that alternative survival pathways compensate for the diminished reliance on BCL2. Thus, despite *in vitro* findings of Nef-induced BCL2 upregulation, *in vivo* HAL tumors may preferentially activate alternative survival pathways such as LMP1-driven NF-κB/PI3K/AKT signaling and MYC amplification, reducing dependency on BCL2 for tumor survival. For instance, EBV may drive oncogenesis via LMP1-mediated activation of NF-κB and PI3K/AKT signaling, thereby providing pro-survival signals independent of BCL2 (22). Additionally, modest upregulation of MYC in HALs may further accelerate cell cycle progression by inducing key regulators such as Cyclin D1, reinforcing the aggressive proliferative phenotype. Collectively, these mechanisms suggest that in HALs, virus-induced survival signals and oncogenic drivers, including MYC, converge to sustain tumor proliferation despite reduced BCL2 expression. This interplay may have significant therapeutic implications, highlighting the potential benefit of targeting alternative survival pathways—such as those mediated by NF-κB/PI3K/AKT or MYC-driven signaling—in the treatment of HALs (23). To improve risk stratification in this heterogeneous patient population, we developed a prognostic model that integrates both traditional clinical indices and HIV-specific factors. Our multivariate Cox regression analysis identified HIV infection (i.e., being in the HALs group), IPI scores, and elevated Ki67 expression as independent predictors of poor overall survival. Several HAL-specific prognostic markers have been reported in the literature. These include high expression of Ki67, EBV positivity, MYC rearrangements, and activation of the NF-κB signaling pathway. Recognition of these markers not only helps in prognostication but also guides therapeutic strategies. Emerging treatments such as EBV-targeted immunotherapies, immune checkpoint inhibitors, and targeted therapies against MYC and NF-κB pathways are under investigation for better management of HIV-associated lymphomas. The prognostic nomogram constructed from these variables demonstrated robust predictive accuracy, with high area under the curve (AUC) values for 1-, 2-, and 3-year survival, and a concordance index of approximately 0.798 upon internal validation with ten-fold cross-validation. Future efforts will include external validation using independent multicenter cohorts to further assess the generalizability and robustness of the prognostic nomogram. Importantly, our model appears to provide enhanced prognostic discrimination compared to established indices such as the IPI, which were primarily developed in HIV-negative populations and do not account for HIV-related immunosuppression or viral oncogenesis. These findings suggest that integrating HIV-specific factors into prognostic models may improve risk stratification in HALs, enabling clinicians to identify high-risk patients who might benefit from intensified or novel therapeutic approaches.

Our analysis also highlights the significant impact of HIV-specific factors—particularly antiretroviral therapy (ART) and immune status—on lymphoma outcomes. In the subgroup analysis of HALs, we found that patients who were receiving ART at the time of lymphoma diagnosis had a significantly higher mortality risk compared with those who initiated ART after diagnosis. However, a longer duration of ART was strongly associated with reduced mortality. This paradox may also reflect lead-time bias, where patients initiating ART at diagnosis likely presented with more aggressive disease requiring immediate therapy, and had less opportunity for immune reconstitution compared to those already maintained on ART. This dual observation suggests that while the initiation of ART at diagnosis may be a marker of advanced HIV disease and poor immune reconstitution, sustained ART over a longer period is associated with better immune recovery and improved survival (14). In essence, long-term ART appears to mitigate the adverse effects of chronic HIV infection by suppressing viral replication, reducing systemic inflammation, and enabling partial restoration of immune surveillance. Conversely, patients who commence ART only at the time of lymphoma diagnosis often have severely compromised immune systems and may have already experienced extensive immune dysregulation, which could predispose them to a more aggressive lymphoma phenotype and poorer treatment outcomes (15). These findings underscore the importance of early HIV diagnosis and prompt initiation of ART, as well as the need to maintain effective long-term viral suppression to optimize clinical outcomes in patients with HALs. Based on these findings, we propose a stratified management approach that integrates both oncologic and virologic considerations for patients with HALs. Our prognostic model provides an effective framework for risk stratification, enabling clinicians to tailor treatment intensity according to each patient’s predicted outcome and HIV status. For those identified as high risk—particularly with unsuppressed viral loads—prompt initiation or optimization of ART is critical to achieve viral suppression as rapidly as possible, while a lower-intensity chemotherapy regimen (e.g., EPOCH) combined with comprehensive infection prophylaxis may mitigate toxicities. In contrast, patients at lower risk and maintaining stable viral suppression can be effectively managed with standard chemoimmunotherapy plus ART, capitalizing on their comparatively preserved immune function (24). By aligning therapy with each patient’s risk profile and ART status, clinicians can maximize survival outcomes, minimize treatment-related complications, and ensure that the oncologic and virologic components of care are addressed in tandem.

Despite these promising findings, our study has several limitations that warrant consideration. The retrospective and single-center design may limit the generalizability of our results, and the relatively small number of HAL cases calls for caution in interpreting some of the subgroup analyses. Detailed treatment data, including specifics of chemotherapy regimens, ART adherence, and supportive care measures, were not fully available, which may confound the observed associations between HIV-specific factors and survival outcomes. The absence of advanced molecular profiling (such as next-generation sequencing to identify genetic rearrangements) limits our ability to explore the underlying mechanisms driving the observed differences in tumor biology. Moreover, the potential gender-specific differences in lymphoma biology and outcomes could not be fully explored due to the sample size; future studies should stratify analyses by gender. Additionally, non-parametric statistical tests were employed to account for the non-normal distribution of clinical and molecular variables, which may influence interpretation. Future multicenter studies with larger cohorts, prospective designs, integrating next-generation sequencing (NGS) and comprehensive molecular analyses are needed to validate our findings and refine the prognostic model further.

## Conclusion

Our study reveals that HIV-associated lymphomas display distinct molecular and clinicopathologic characteristics, including a high rate of EBV infection, a predominant GCB phenotype, and a paradoxical profile of high proliferative activity coupled with low BCL2 expression. These features likely result from the interplay between chronic HIV-induced immunosuppression and virus-driven oncogenesis, leading to a more aggressive tumor phenotype and poorer survival. Our integrated prognostic model, which incorporates both traditional clinical indices and HIV-specific factors, offers enhanced risk stratification and has the potential to guide personalized therapeutic strategies. Moreover, the critical impact of ART duration on survival underscores the importance of effective long-term HIV management in improving lymphoma outcomes. Moving forward, a multidisciplinary approach that optimizes both lymphoma-directed therapy and HIV control is essential. Tailored treatment strategies, informed by robust prognostic models and comprehensive molecular profiling, hold promise for improving the survival and quality of life of patients with HALs.

## Data Availability

The original contributions presented in the study are included in the article/**Supplementary Material**. Further inquiries can be directed to the corresponding authors.
