# Supplementary material for: Molecular and clinical insights into HIV-associated and HIV-negative aggressive B-cell lymphomas: prognostic quantitative biomarker analysis and therapeutic implications
Source: Front Oncol. 2025 May 27;15:1603801. doi: 10.3389/fonc.2025.1603801 (PMC12148868; doi:10.3389/fonc.2025.1603801)
Supplement: Supplementary file 1 [file DataSheet1.docx]

**Supplementary Table 1. Primary Antibodies Used for IHC and ISH Staining**

| Antibody | Manufacturer | Clone | Dilution | Instrument |
| --- | --- | --- | --- | --- |
| Ki-67 | ZSGB Biotech, China | UMAB107 | RTU | Benchmark XT, Roche |
| c-myc | ZSGB Biotech, China | OTIRIH2 | RTU | Benchmark XT, Roche |
| BCL2 | ZSGB Biotech, China | LN22 | RTU | Benchmark XT, Roche |
| BCL6 | MAXIN Biotech, China | EP121 | RTU | Benchmark XT, Roche |
| MUM1 | MAXIN Biotech, China | MUM1P | RTU | Benchmark XT, Roche |
| CD10 | MAXIN Biotech, China | MX002 | RTU | Benchmark XT, Roche |
| EBER | ZSGB Biotech, China | ISH-7001 | RTU | Benchmark XT, Roche |

RTU indicates ready to use

**Supplementary Table 2. HR (95%CI) and P-values for Univariate, Multivariate, and Stepwise Cox Regression Analysis of Prognostic Factors in lymphoma patients**

| Characteristic | Univariate | | Multivariate | | Cox (Stepwise Selection) | |
| --- | --- | --- | --- | --- | --- | --- |
|  | HR (95%CI) | *P* Value | HR (95%CI) | *P* Value | HR (95%CI) | *P* Value |
| HIV | 2.13 (1.26 – 3.49) | 0.004 | 1.85 (1.08 - 3.18) | 0.026 | 1.77 (1.05 - 3.01) | 0.034 |
| Sex | 3.11 (1.58 – 6.13) | 0.001 | 2.37 (1.20 – 4.64) | 0.013 | 2.36 (1.20 – 4.64) | 0.013 |
| Age | 1.47 (0.84– 2.57) | 0.177 |  |  |  |  |
| LDH | 2.57 (1.41– 4.67) | 0.002 | 1.06 (0.54 – 2.08) | 0.862 |  |  |
| Stage | 3.84 (1.63 – 9.07) | 0.002 | 0.57 (0.19 – 1.72) | 0.321 |  |  |
| IPI | 6.80 (3.04 – 15.22) | <0.001 | 12.85 (3.90 -41.97) | <0.001 | 9.35 (4.20 – 20.82) | <0.001 |
| Classification | 0.84 (0.24 – 3.00) | 0.789 |  |  |  |  |
| Nodal | 0.96 (0.58 – 1.59) | 0.868 |  |  |  |  |
| Ki67_AOD | 12.58 (1.52- 104.1) | 0.019 | 3.03 (0.85 – 9.75) | 0.087 | 3.04 (0.85 – 10.85) | 0.087 |
| Ki67_Area | 1.39 (1.00 – 1.94) | 0.050 |  |  |  |  |
| HBV | 0.92 (0.55 – 1.57) | 0.770 |  |  |  |  |
| EBER | 1.03 (0.61 – 1.73) | 0.925 |  |  |  |  |

**Supplementary Table 3. HR (95%CI) and P-values for Univariate Cox Regression Analysis of Prognostic Factors in HIV+ lymphoma patients.**

| Variable | HR | 95% CI | *P* Value |
| --- | --- | --- | --- |
| ART Duration | 0.289 | 0.186 – 0.449 | 3.34e-08 |
| ART Status at Diagnose | 4.718 | 1.523 - 14.612 | 0.00715 |
| CD4 Count | 1.040 | 0.755 – 1.433 | 0.81061 |
| EBER | 1.811 | 0.758 -4.328 | 0.18144 |
| Ki67_AOD | 1.551 | 0.156 – 15.431 | 0.70808 |
| IPI | 1.890 | 0.564 – 6.327 | 0.30208 |
| Sex | 2.068 | 0.233 – 18. 381 | 0.51467 |


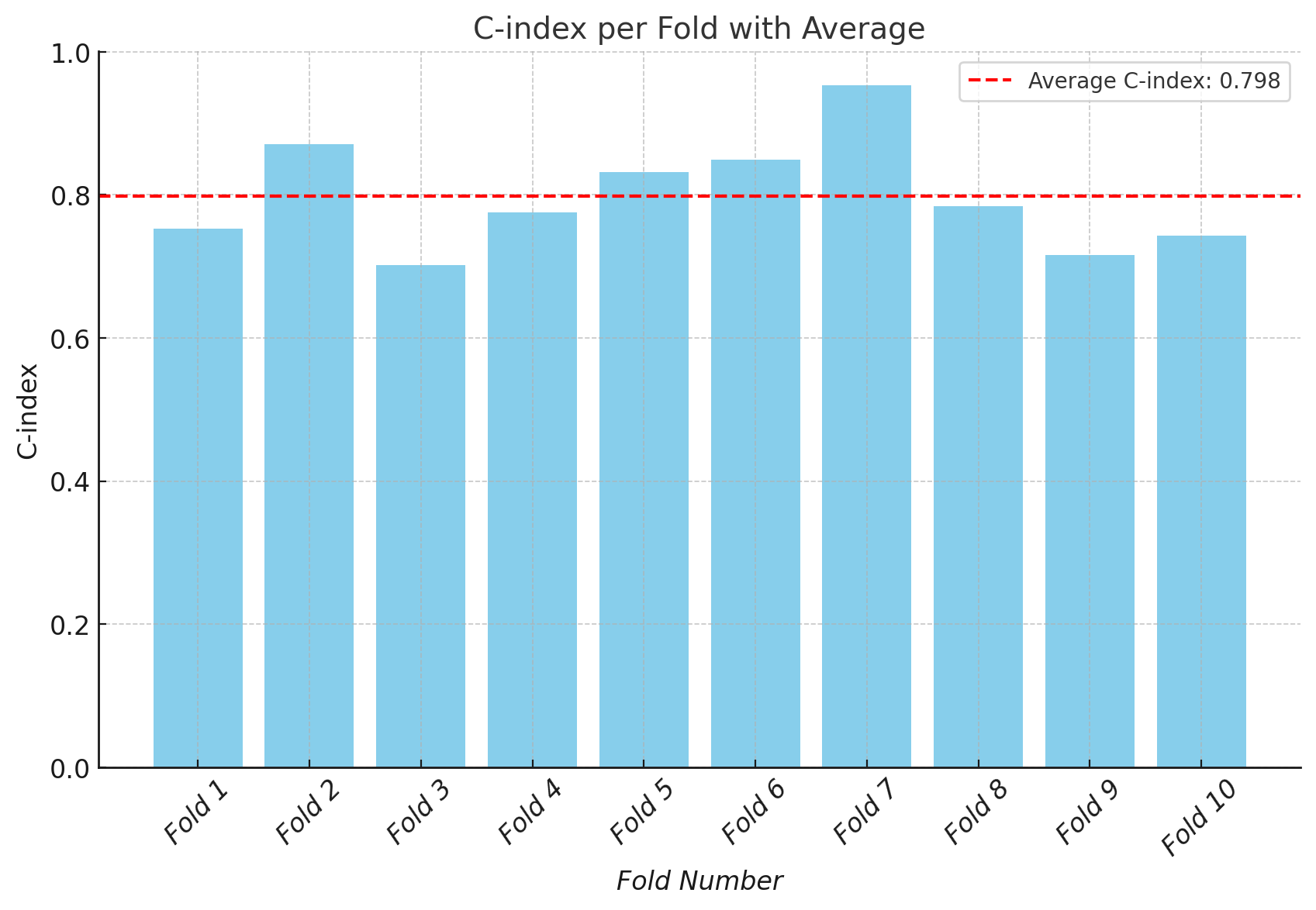


**Supplementary Figure 1. C-index per fold with average.**

The bar chart shows the C-index for each fold in a cross-validation analysis. The red dashed line represents the average C-index (0.798) across all folds.
